# Collective Rhythm as an Emergent Property During Human Social Coordination

**DOI:** 10.3389/fpsyg.2021.772262

**Published:** 2022-02-10

**Authors:** Arodi Farrera, Gabriel Ramos-Fernández

**Affiliations:** Mathematical Modeling of Social Systems Department, Institute for Research on Applied Mathematics and Systems, National Autonomous University of Mexico, Mexico City, Mexico

**Keywords:** interpersonal coordination, collective rhythm, emergence, spontaneous mimicry, synchronization

## Abstract

The literature on social interactions has shown that participants coordinate not only at the behavioral but also at the physiological and neural levels, and that this coordination gives a temporal structure to the individual and social dynamics. However, it has not been fully explored whether such temporal patterns emerge during interpersonal coordination beyond dyads, whether this phenomenon arises from complex cognitive mechanisms or from relatively simple rules of behavior, or which are the sociocultural processes that underlie this phenomenon. We review the evidence for the existence of group-level rhythmic patterns that result from social interactions and argue that the complexity of group dynamics can lead to temporal regularities that cannot be predicted from the individual periodicities: an emergent collective rhythm. Moreover, we use this interpretation of the literature to discuss how taking into account the sociocultural niche in which individuals develop can help explain the seemingly divergent results that have been reported on the social influences and consequences of interpersonal coordination. We make recommendations on further research to test these arguments and their relationship to the feeling of belonging and assimilation experienced during group dynamics.

## Introduction

The Mexican wave (hereinafter the wave) propagates in stadiums through the action of successive groups of fans who briefly stand up with their arms up (see Farkas et al., 2002). To anyone who has been part of it, it is evident that it arises from a small group of initiators, that no individual fan has control over its development, and that it expands spontaneously from the interaction between fans following a simple local rule such as “if the person next to you stands up, you stand up; if they sit down, you do so as well.” In contrast, when seen from a distance, this collective behavior seems to have a life of its own, or a dynamic that does not easily correspond to the individual behaviors that start and sustain it throughout the stadium and whose development is not controlled by any agent or external factor. Coordinated activities like this are examples of *self-organizing emergent phenomena* that arise from and are sustained by the collective in non-intuitive ways (i.e., weak emergence: Bedau, 1997).

A dyadic interaction is a smaller scale and widely studied example of this type of self-organizing phenomenon. During social activities, participants not only spontaneously coordinate at the behavioral but also at the physiological and neural levels (Hoehl et al., 2021). This coordination organizes the biological rhythms of the individual (Feldman, 2012) and gives a temporal structure to the interpersonal dynamics. Importantly, in humans this phenomenon may signal affiliation and has been associated with prosocial behaviors (Gordon et al., 2020) and, indirectly, with the feeling of belonging and assimilation to the group that people experience when participating in collective activities such as rituals (Mogan et al., 2017). Although research on this topic has gradually shifted toward the study of interactions of more than two people and of more spontaneous, everyday activities, it is still not clear, for example, how the rhythmic patterns observed in group activities are related to the said sociocultural processes or if their characteristics are similar to those observed in dyads. Particularly, in the latter case, it remains to be addressed whether the temporal organization of a group activity is an example of self-organization emerging, as in the case of the wave, from the individual rhythms.

Examining whether a collective temporal pattern is an emergent phenomenon is important for our understanding of social complexity and cognition (see Boyer and Ramos-Fernandez, 2018). First, this knowledge could be used to explore the extent to which this temporal organization arises from complex cognitive mechanisms or from relatively simple rules of behavior, such as those required for the wave. Second, the study of emergent collective rhythms could inform us about the cognitive capacities supporting the perception of group movement (e.g., Cracco et al., 2021) or those associated with rhythm perception and production (Ravignani et al., 2014). Finally, it could be used to complement our understanding of sociocultural practices such as collective effervescence (Xygalatas et al., 2011) or musical improvisation (Walton et al., 2015).

In this contribution, we put forward the hypothesis that collective rhythms emerge during naturalistic interactions, as the basis for further research in this area. We review some of the evidence that supports this hypothesis and the relationship of macroscopic phenomena with the social processes that have been associated with them. We begin by proposing a definition of collective rhythm, and then review two mechanisms that have been frequently used to explain patterns, both in time and form, of interpersonal coordination: behavior matching and interactional synchrony (Bernieri and Rosenthal, 1991). We then propose a framework on how these mechanisms can be used as minimal explanations of the temporal organization of social interactions at the individual and group levels, and how taking their social impact into account can help explain seemingly divergent results on their functional significance in the literature. The final section integrates the evidence to explore whether coordinated activities could lead to the emergence of a collective rhythm, its relationship to the group feeling of belonging and connection, and if such phenomena could be integrated into the construction of the human niche.

### Definition of Collective Rhythm

The organization of behavioral events and its development over time build up the temporal structure of behavior (e.g., Ravignani et al., 2014). In a single individual, complex behaviors are temporally structured by layers of multimodal signals (Pouw and Dixon, 2020) nested on different time scales (Abney et al., 2021). For example, several occurrences of body movements and utterance activity produced at short scales (e.g., typing) can be grouped into larger time scales, in turn delimited by moments of no activity (e.g., writing a chapter of a manuscript in short bursts). Likewise, in even larger time scales, such ensembles of behavioral activity can be arranged in clusters depending on the constraints and contingencies of the various stages of the task at hand (e.g., planning, writing, evaluation). This type of temporal pattern (i.e., the duration and timing of events) of the nested organization of any series of behavioral or physiological activity builds the rhythm of the individual (Ravignani and Norton, 2017).

**FIGURE 1:**
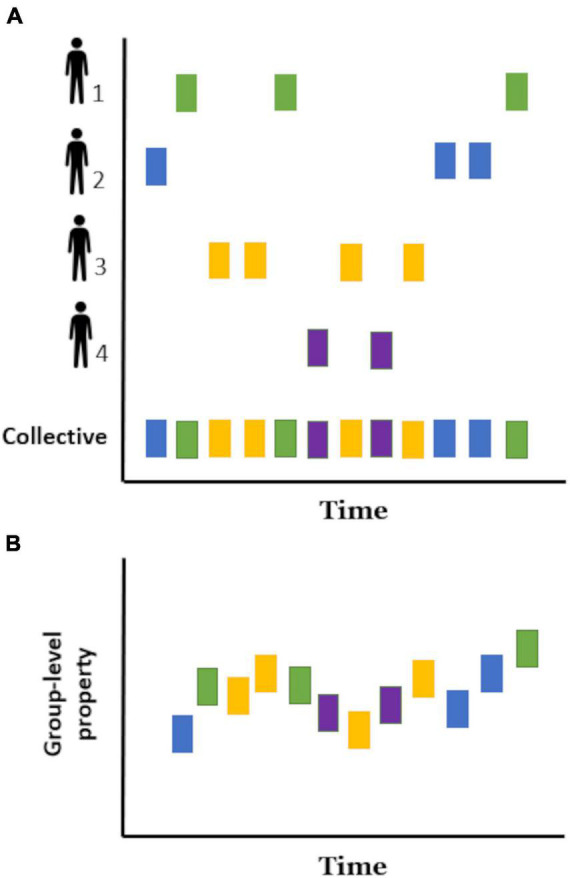
Schematic representation of temporal rhythmic patterns in a group of four individuals. The onset time of a given behavioral event (e.g., an utterance) is represented as a rectangle with each color corresponding to a different individual. **(A)** Temporal patterns of behavior recovered from each of the four individuals (individual temporal patterns) and from all participants (collective temporal pattern). **(B)** Emergent collective rhythm: a collective temporal pattern that cannot be predicted from the individual periodicities alone. In this case, the vertical axis represents a new group-level property.

When several people are involved in a joint behavioral event (chorus behavior, sensu Ravignani et al., 2014), the temporal pattern of each individual reflects the dynamics of their interaction (Figure 1A), in the sense that participants reciprocally adjust their behaviors to the actions and reactions of others and the environment in which they interact. At one end, a group in which each individual behaves independently of the others (for example, tossing a coin and taking a step to the left or the right depending on whether it is heads or tails), will have disconnected temporal patterns. At the opposite end, a group in which its participants influence and are influenced by other participants’ behavior will show a varying degree of temporal organization depending on the individual and collective constraints and contingencies of the task in question. For example, the temporal structure will vary whether people sing in unison (e.g., synchrony), coordinate into subgroups to build a Lego structure (e.g., complementary) or enter a roundabout (e.g., alternating).

In longer timescales, these individual temporal patterns and the joint dynamics of the interaction can change over time (Wiltshire et al., 2018). Some common examples of this evolution are the temporal patterns of body movement during music improvisation, the steps of pedestrians passing over a footbridge and the swing of metronomes placed on a moving surface, which start out as decoupled patterns (i.e., independent) and eventually fall into coordinated behaviors (e.g., simultaneous or alternating) because the medium in which they interact (i.e., music, shaking surface) couples them weakly.

The timing and duration of behavior events recovered from all participants at the same time (Figure 1A) can be used to describe the rhythm of a given group. However, as mentioned above, it is still unknown to what extent and under which circumstances these collective rhythms are emergent self-organizing temporal patterns. Then, in order to facilitate its study, we define an *emergent collective rhythm* (Figure 1B) as the temporal patterns of behavior arising in groups that cannot be predicted from the individual periodicities alone. These patterns can be thought of as a group-level property, in this case, a rhythm “with a life of its own,” analogous to the wave observed in crowded stadiums. Moreover, similar to the rhythm in music, this group-level phenomenon could be a temporal pattern with which individuals could coordinate. However, in contrast to a prespecified rhythm provided by a musical sheet (i.e., an external controlling component), the collective rhythm would both influence and be influenced by the individual rhythms and contingencies of the interaction. This is not to say that musicians do not take part in instances of emergent collective rhythm, given that music improvisation is a great example of this group phenomenon.

Different emergent forms of social coordination have been reported (spontaneous coordination: Knoblich et al., 2011; synergy: Fusaroli et al., 2014; self-similarity: Abney et al., 2021), but the study of this emergence in the context of rhythmic patterns has been carried out mainly in dyads and on short timescales. However, this approach disregards that, over time, the specific arrangement of interactions between participants and between them and the environment can produce dynamics that are not easily predictable (Page, 2015). Moreover, it overlooks that once formed, group-level phenomena can constrain the evolution of the individual components and produce even more complex dynamics (i.e., downward causation: Flack, 2017). In both cases, this path dependency would mean that the outcome of a coordination dynamic, either in terms of the resulting temporal pattern or its social consequences, is contingent on what happens during its evolution. And in such circumstances, focusing only on certain moments of the interaction and ignoring its change over time may lead to incorrect inferences. Therefore, the study of emergent collective rhythms also needs to consider these dynamics over time as well as the possibility of downward causation from the macroscopic to the microscopic level.

## Mechanisms

In this section we review two mechanisms that have been frequently used to explain coordination dynamics: behavior matching and interactional synchrony. We chose these mechanisms as the simplest explanation of how behavior can spontaneously be organized: people unintentionally mimic (Lakin and Chartrand, 2003) and synchronize (Knoblich et al., 2011) their behavior with others. Furthermore, we chose these mechanisms because they have been studied in species other than humans. This evolutionary perspective seeks to complement previous accounts of interpersonal coordination based on processes observed solely in contemporary humans (e.g., behavioral alignment: Rasenberg et al., 2020, interpersonal synergies: Fusaroli and Tylén, 2016, coordination: Clayton et al., 2020), and to encourage its use to compare different systems of animal behavior.

### Behavior Matching

People involuntarily imitate the movements and facial expressions of others. Although the terminology used to explain this phenomenon depends on the domain (Genschow et al., 2017), here, the concept of *behavior matching* encompasses both the study of spontaneous mimicry and automatic imitation.

When coordinating with others, we observe a tendency to spontaneously adopt the behaviors of interaction partners (Lakin et al., 2003), even if it affects the efficiency of the observer’s own movements (Forbes and Hamilton, 2017). Importantly, this automatic tendency to imitate is fundamental in contexts in which participants need to predict others’ movements (Sacheli et al., 2015; Era et al., 2020), suggesting that automatic imitation is associated with action prediction during interpersonal motor coordination.

Behavior matching can occur from fractions of a second (e.g., finger mimicry) to several seconds (e.g., yawning) after the stimulus (Prochazkova and Kret, 2017; Arnold and Winkielman, 2019). And in scenarios in which individuals automatically imitate the actions of multiple agents (Cracco et al., 2015), the response time decreases asymptotically when the observed movements were congruent and increase linearly on incongruent ones (Cracco and Brass, 2018).

Spontaneous mimicry is based on motor resonance, where the action of observing others activates neurons that represent that same action in the observer’s motor system (cf. Uithol et al., 2011, on controversies in the interpretation of motor resonance). This phenomenon is thought to allow for a quick communication with other members of the group about important aspects of the physical and the social environment (e.g., physiological internal states like arousal due to food availability or fear due to predator presence). For instance, in different species of mammals, mimicry has been found to be associated with socially relevant facial displays, such as the play face (Palagi et al., 2020), which is assumed to communicate a playful mood (Palagi et al., 2019) even when phylogenetically distant species play together (e.g., Maglieri et al., 2020). Motor resonance is also communicative because it affects how individuals perceive their surroundings. For example, in a series of experiments, Fini et al. (2017) showed that extra-personal space representation is a function not only of the individual’s motor potential but also of the bodies, motion, and intentions of other individuals. In addition, it has also been proposed that spontaneous mimicry underlies emotional contagion, whereby the perception of another’s emotional state automatically activates the same neural response (including the mirror neuron system) of the affective state in the observer, along with corresponding somatic and autonomic responses (Xavier et al., 2016; Palagi and Scopa, 2017; Prochazkova and Kret, 2017).

By creating similarity between participants and by providing a basis for inferring other’s emotions, both behavior matching and emotional contagion are linked to empathy, rapport, and prosocial behavior (see Lakin et al., 2003). These prosocial consequences facilitate interactions and coordination of common goals, for example, by conveying the readiness for coordinated action (e.g., playful interactions: Palagi and Scopa, 2017; music and dance: Phillips-Silver and Keller, 2012), by smoothing the interaction and making it low-maintenance (see Lakin, 2013), or by communicating the social role of the interactants (e.g., dominance in primates: Palagi et al., 2020).

Despite the potential role of spontaneous mimicry in social interactions, its ontogeny has been little explored. We know, for example, that mimicry of facial expressions is present in neonates (Palagi and Scopa, 2017, but see Oostenbroek et al., 2016), and that in infants, it occurs in response to multimodal, not unimodal information (visual or auditory separately, Isomura and Nakano, 2016), and to happy and fearful faces as opposed to angry faces (Kaiser et al., 2017). Additionally, the tendency to mimic is positively related to the amount of facial imitation received from the caretaker (Klerk et al., 2018) and modulated by the quality of early attachment relationships (Vacaru et al., 2020). Later in life, individuals mimic more smiling faces than angry, fearful or sad faces (Sachisthal et al., 2016), and are more aroused at pleasant, but not unpleasant facial expressions (Fujimura et al., 2010). The mimicry response can be further influenced by the *a priori* levels of empathy of the mimicker (Rymarczyk et al., 2016), and of liking for the person being mimicked (Stel et al., 2010).

Overall, the individual mimicry response has been shown to be a stable individual trait (Hess et al., 2016) reduced in the absence of social utility (Beffara et al., 2012) and modulated by social cues about the type of task performed (Arnold and Winkielman, 2019; Era et al., 2020) or group membership (Sacheli et al., 2015; van Schaik and Hunnius, 2016; de Klerk et al., 2019) even in different mammalian species (Palagi et al., 2020). Furthermore, studies on power dynamics in humans have shown that spontaneous mimicry not only follows simple direct-matching rules (e.g., smile to a smile) but that social cues like hierarchy can lead to the counter-mimicry or opposite matching (Arnold and Winkielman, 2019; Palagi et al., 2020), such as when high-power perceivers smile in response to angry expressions of other high-power targets (Carr et al., 2014).

In sum, spontaneous mimicry of others’ behavior is an important component of interpersonal coordination that facilitates the interaction and promotes affiliation.

### Interactional Synchrony

During social interactions, we not only observe an automatic imitation of perceived behaviors, but also that individuals are able to anticipate other’s behaviors and align the timing of their movements accordingly. Integrating the evidence on this ability is complicated because the terminology used to refer to it changes depending on the discipline. We chose the concept of interactional synchrony (Bernieri and Rosenthal, 1991) to encompass this variability because it includes two broad subfields: the study of *entrainment* and of *synchronization*. Although both deal with the temporal coordination of two or more events (Bittman, 2021), the first one focus on the ability of an individual’s endogenous rhythms to entrain to time cues at a variety of phase angles, while the second with the ability of an individual’s locomotor rhythms to align in phase to a given time cue. Therefore, we use the term entrainment only to refer to endogenous rhythms and synchronization everywhere else.

In the two sections below, we focus separately on endogenous rhythm and its entrainment and on spontaneous motor tempo (SMT) and its synchronization. We review studies that complement previous accounts of the production and perception of rhythm in music and speech (Ravignani et al., 2017), and that emphasize the potential role of the plasticity and development of the individual’s internal rhythms in our ability to recognize and synchronize with the rhythms of others. In particular, we highlight that in both cases the flexibility and propensity to which individuals coordinate with others is modulated by the experience they have during development (e.g., parental coordination strategies, musical training) and the emotional context of the interaction.

#### Endogenous Rhythms

Endogenous rhythms (Kriegsfeld and Nelson, 2009) are ubiquitous in nature and are assumed to be the organism’s adaptation to the highly predictable and cyclic environment that results from physical forces (e.g., the light-dark cycle - LD-, the seasons). They are regulated by the organism’s biological clock and have cycles in the millisecond-to-year range. Importantly, endogenous rhythms are self-sustained and can synchronize with rhythmic signals, i.e., they will continue to cycle in the absence of any time cue but will actively entrain in the presence of one. Entraining favors the alignment of behavioral and physiological rhythms to those of the environment: for example, instead of responding to the immediate food availability, organisms keep track of time internally, which allows them to anticipate changes in such resources throughout the day or year and to respond accordingly, even when temporal cues are unavailable (e.g., in caves) or misleading (e.g., light exposure in urbanized environments) (Helm et al., 2017).

In social species, activities to be performed together with conspecifics (e.g., foraging, mating) also form part of the temporal layout of the environment, i.e., the arrangement of biotic and abiotic rhythms whose different periodicities are overlapping in the environment and with which organisms can align. Failure to keep up with these activities could make an individual more susceptible to predation or ostracism. Accordingly, social stimuli have been shown to entrain endogenous rhythms (Mistlberger and Skene, 2004; Favreau et al., 2009; Bloch et al., 2013) both in species with limited access to the main environmental time cue, i.e., the LD cycle, or those living on natural LD cycles. For example, the circadian synchronization of marmosets placed in temporal isolation (i.e., constant light condition) is favored by the activity profile (Melo et al., 2013) or vocalizations (da Silva et al., 2014, but see Erkert et al., 1986) of conspecifics, or acoustic and olfactive contact between reproductive pairs (Bessa et al., 2018). In humans, the evidence suggests that social signals are weaker than light cues, but both jointly influence the circadian response (i.e., rhythms with cycles of approximately 24-h) (Davidson and Menaker, 2003; Mistlberger and Skene, 2004).

Evidence for the role of endogenous rhythms in social coordination is found in studies addressing the mother-infant attunement, essential to the offspring’s survival (Harrist and Waugh, 2002). In these studies, synchrony facilitates the coordination of hormonal, physiological, and behavioral cues into an affiliative bond that facilitates individuals’ physiological regulation during development. For example, we know that fetal rhythms engage with the LD cycle indirectly through maternal signals (e.g., body temperature), that the mother’s heart rate or walking pace facilitate the infant’s physiological regulation (Bobin-Bègue, 2019), and that during face-to-face interaction, vocal and affect exchanges increase the degree of physiological linkage (Feldman, 2012). Moreover, such physiological dynamics have been associated with emotion regulation and empathy levels later in life (Lee et al., 2017; Levy et al., 2019; Levy and Feldman, 2019).

Overall, this literature shows that individuals in many animal species, including humans, are capable of processing rhythms and synchronizing with the immediate social environment even before birth. By underpinning the child’s social and emotional growth, this ability is in turn crucial in shaping the adult’s ability to coordinate and relate to others.

#### Spontaneous Motor Tempo

Throughout life, we regularly carry out rhythmic locomotor activities (e.g., infant’s spontaneous sucking, walking) that show a self-sustained repetition rate and the ability to synchronize with rhythmic signals, i.e., we all have a preferred pace to perform them, but we can synchronize them with an external time cue with a different pace (e.g., clapping to the beat of a song). This preferred, “internal,” “natural” tempo or SMT is speculated to reflect the intrinsic tempo of a spinal central pattern generator (MacDougall and Moore, 2005). It has been studied mostly by using a tapping task where participants are asked to tap their hands on a table at a comfortable rate (McAuley, 2010), showing that SMT is an individual trait that develops throughout ontogeny, becoming slower and more stable in adulthood (McAuley, 2010; Bobin-Bègue, 2019; Monier and Droit-Volet, 2019). Even when different movements within an individual have different tempi (e.g., Qi et al., 2019), there is a preference for 500 ms periods, either during everyday locomotor activity or activities performed in laboratory conditions (MacDougall and Moore, 2005).

Humans and a few other species (Wilson and Cook, 2016) can synchronize these motor tempi with external temporal signals. Particularly, humans are able to adapt to tempi that are different from their SMT, either deliberately as in tapping tasks (Repp and Su, 2013) or spontaneously, as when neonates modify the tempo of their sucking (Bobin-Bègue et al., 2006) or stepping (Provasi et al., 2014) according to a rhythmic stimulus, or when music engages infant’s movements (Zentner and Eerola, 2010) or the walking pace of adults (Buhmann et al., 2016). Studies using a variation of the tapping task show that flexibility in synchronization to different tempi develops with the maturation of the neuromuscular system during the first years of life, that it reaches its adult form at about 8–10 years old (Provasi and Bobin-Bègue, 2003; Rocha and Mareschal, 2016), and that its accuracy can be improved by extensive training. Indeed, musicians synchronize more flexibly across tempi than non-musicians (Scheurich et al., 2018) but even after this rigorous training, the spontaneous rates at which they perform naturally (i.e., SMT) remain stable (Zamm et al., 2018). Simultaneously, this flexible response to new tempi and its accuracy are also influenced by the individual’s internal tempo. During synchronization tasks, musicians with a more stable tempo are more synchronous across different tempi (Scheurich et al., 2018), while synchronization accuracy increases when the external cue is close to the individual’s SMT (Loehr and Palmer, 2011).

Although the above characteristics make the internal motor tempo a potential key coordinating mechanism for social interactions (Jungers et al., 2002), there is little research on the role of its flexibility within a social context. On the one hand, we know that the individual’s tempo influences the accuracy in the timing with others. For example, a similar SMT between participants when walking side by side facilitates synchronization to the other’s movements (Repp and Su, 2013). Likewise, musicians with matching spontaneous rates of solo performance show greater synchrony than mismatched partners (Jungers et al., 2002; Loehr and Palmer, 2011; Palmer et al., 2019). On the other hand, the social environment may influence the accuracy of such synchronization. For instance, although children as young as 2.5 years are unable to synchronize with acoustic pulses, those that drum together with an adult can do so with high accuracy, as opposed to when drumming along an audio-visual or an acoustic stimulus (Kirschner and Tomasello, 2009). In addition, children’s previous everyday experiences and the musical practices within their culture could also influence whether a child will spontaneously synchronize with the experimenter and the accuracy of this task (Kirschner and Ilari, 2013). Moreover, the previous experience of the infant’s own body movement (i.e., being moved up and down to a beat) plays an important role in their rhythm perception and listening preferences (Phillips-Silver and Trainor, 2005).

We also know that its ability to adapt allows the internal tempo to modulate and be modulated by others’ tempi from very early in development. For example, infants move their limbs in coordination with the speech behavior of adults (Condon and Sander, 1974) while adults change their speech rate according to infants’ linguistic competence (Narayan and McDermott, 2016). Finally, we know that despite being a relatively stable trait, the individual’s internal tempo may vary depending on the emotional context of the interaction, and that this will affect its ability to synchronize with a beat (Monier and Droit-Volet, 2016).

Summing up, during interpersonal coordination, the rhythms (i.e., endogenous, SMT) of different individuals interact in a shared temporal structure. Given that these rhythms can be synchronized with temporal cues with great flexibility and accuracy, they are potentially key to such integration, affecting and being affected by social exchanges. They represent stable individual differences in the pace of periodic movements, and, at the same time, encompass the fluctuations that individuals experience due to, for example, the emotional context of an interaction. During activities that follow tempi with a high degree of consistency (e.g., music), the synchronization is more accurate when the task’s tempo is closer to the tempo of the participants. Whereas during activities that are not constrained by a prescribed tempo, that is, those in which no agent controls the development of the dynamic (e.g., face-to-face interaction), a certain degree of flexibility may facilitate the mutual modulation.

## Bodily States and the Temporal Pattern of Behavior

An emergent group-level phenomenon depends strongly on the specific way in which its components combine and interact (Page, 2015). Therefore, in this section we emphasize how spontaneous mimicry and synchrony contribute to the integration of temporal patterns during social interactions at the individual (i.e., bodily state) and group level (i.e., collective temporal pattern), and how this could lead to an emergent collective rhythm. The assumptions underlying this argument are presented in a simple causal model (Rohrer, 2018) below.

**FIGURE 2:**
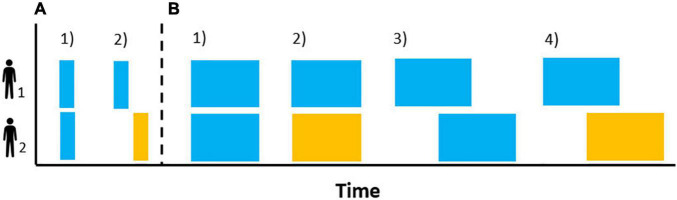
Schematic representation of discrete **(A)** and continuous **(B)** patterns of observed behaviors at the level of the interaction. Behavior matching of the same behavior without lag [**(A1)** person matching the smile of the other interactant] or with lag [**(A2)** listening to another yawn activating the listener’s yawn]. Synchrony of the same behavior [**(B1)** clapping together] and different behavior [**(B2)** the infant’s arm movements matching in time and intensity the mother’s voice], both matching in time. Synchrony of the same [**(B3)** the pitch of one person matching the pitch contour of the previous intervention] and different [**(B4)** the rhythm of someone’s nod matching with a slight offset the speech rhythm of another] behaviors with lagged timing.

According to the evidence presented so far (see section “Mechanisms”), behavior matching and interactional synchrony are key in organizing the temporal structure of an interaction. On the one hand, either as a sequence of discrete events between individuals or as the onset of coordinated behaviors between them (e.g., Casetta et al., 2021), behavior matching (Figure 2A) marks the onset of a given behavior and, thus, builds its temporal pattern (see Figure 1). On the other, given that the individual rhythms (i.e., endogenous, SMT) can be synchronized with temporal cues with great flexibility (i.e., varying phases) and accuracy, interactional synchronization (Figure 2B) could organize the temporal patterns of different participants by reducing the rhythmic diversity between them to a single rhythm, to alternate rhythms or by producing rhythms coupled to varying degrees. Contingencies and constraints at this individual level will create variability in the outcome observed for each mechanism and thus influence the social dynamics. For instance, *a priori* levels of liking to the other participants will change an individual’s tendency to mimic others (Stel et al., 2010) while an individual’s internal tempo will affect the accuracy with which they synchronize to others’ temporal cues (Loehr and Palmer, 2011).

**FIGURE 3:**
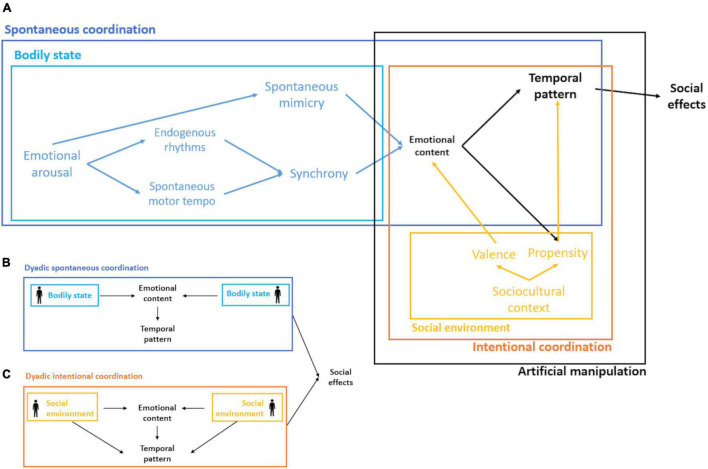
Schematic representation of interpersonal coordination and its social effects. **(A)** Bodily state: depicts the intra-individual variation due to changes in emotional arousal affecting the individual’s propensity to mimic and synchronize; the latter mediated by the individual’s rhythms (e.g., endogenous rhythms and spontaneous motor tempo). Spontaneous coordination arises, at least in part, from the accommodation of bodily states between interactants. Social environment refers to the context in which individuals develop, which influences, for example, their propensity to participate in coordinated activities or the social valence that define prosocial or antisocial behavior. Intentional coordination considers the influences of the social environment at the interaction level. Artificial manipulation includes instances in which intentional coordination is artificially manipulated. **(B)** Dyadic spontaneous coordination: the exchange of bodily states between interactants influences, at the interaction level, the dynamics of emotional content and the temporal pattern of behavior. **(C)** Dyadic intentional coordination: like panel **(B)** but with an additional influence of the social environment on the temporal pattern, for example, when the goal of a task (e.g., to compete or cooperate) and the participants’ social structure constraints the set of observed behaviors during the interaction.

In practice, behavioral matching and synchronization are intertwined and often used interchangeably. One example of their tangled expression has been observed in studies on our ability to process temporal information. According to this research, although our internal clock allows us to accurately estimate time, its representation can be distorted by the physiological activation of emotional arousal (see Droit-Volet, 2019**:** 112). At the same time, spontaneous mimicry of emotional facial expressions activates such physiological arousal, thus potentially influencing the perception of time. Accordingly, the perception of another’s emotional face distorts the accuracy of the estimate of the duration of the presented stimuli, but there is no temporal distortion if facial mimicry is inhibited by asking the participants to hold a pen between their lips (Effron et al., 2006). These results suggest that during an interaction both phenomena might entail a labile response (Figure 3A: bodily state) that appears to be integrated in the organism, particularly in situations with affective content (e.g., parent-infant interaction). In other words, contingencies at the interaction level, like those related to its affective context, may promote variability in this labile response. For instance, paying attention to an audiovisual stimulus (e.g., listening to the same story: Pérez et al., 2021) leads to spontaneous synchronization (Figure 3: spontaneous coordination) between participants, but the extent of this coordination depends on the emotional content of such stimulus and the existent social relationships between the individuals (Bizzego et al., 2020). Another example of how these interactions promote non-intuitive or easily predictable outcomes is the study of Gleibs et al. (2016). This study shows that the effect of group membership on automatic imitation between participants depends on the expected goal of the interaction. More precisely, when the goal is to compete, participants imitate to the same extent an ingroup than an outgroup target. Conversely, for cooperation, group membership is important: participants imitate more an ingroup than an outgroup target.

Likewise, the cultural niche in which individuals interact and develop produces constraints and contingencies that affect the development of a given social interaction. We know that organisms not only adapt to the environment, but that by transforming it, they modify the selection pressures that act on themselves through the process of niche construction (Laland and O’Brien, 2011). Humans modify their environment mainly through cultural processes, which impacts developmental processes and the traits that are considered as adaptive within a given population at a given point in time. Individuals will differ in their propensity to coordinate bodily states with others or the activities that trigger such coordination because of growing up in different culturally constructed environments (Figure 3A: social environment), or due to differences in the way in which cooperation, or empathy are encoded within the sociocultural context, different norms or cultural preferences, or differences in the social valence associated with the activities that are related to prosocial or to antisocial effects. For instance, even though children synchronize their drumming with higher accuracy to that of a human partner than to an external acoustic stimulus (Kirschner and Tomasello, 2009), Brazilian children were reported to do so more spontaneously and with greater accuracy than German children (Kirschner and Ilari, 2013). Importantly, the authors found that these differences were partially explained by differences in children’s active musical practice at home. These results may suggest that the culture’s practice (i.e., the constructed niche) in which the individual develops modulates the tendency to coordinate with others but not necessarily affects the individual ability *per se*. In other words, given a similar cultural environment, both groups of children would not differ on average in the accuracy and propensity to coordinate with others. Thus, the influence of the social environment on interpersonal coordination (Figures 3A,C: intentional coordination), could be incorporated not directly through mimicry and synchrony, but through the emotional content and the type of temporal pattern unfolded at the interaction level due to cultural encodings. Although there are no systematic reviews of cross-cultural differences in this regard, both spontaneous mimicry and synchronization have been reported to be sensitive to sociocultural cues, such as social status (Boukarras et al., 2021), group membership (Sacheli et al., 2015; Palagi et al., 2020) or the degree of competition of the task (Spapé et al., 2013; Era et al., 2020).

All these constraints and contingencies will feed back and influence whether during an interaction one participant adapts to the changes of others (unidirectional: Demos et al., 2017) or whether participants adapt and respond to one another (bidirectional: Lorenz et al., 2014). Nevertheless, while the former requires one participant to do all the adapting, the latter gives the participants an opportunity to modulate each other’s bodily states and opens up the possibility of a new, more stable form of interaction that is different from the initial behavior of each individual, i.e., an emergent organization (Shockley et al., 2009). An example of such emergence would be the affective responses of patients and therapists, which stabilize around a homeostatic balance (Koole and Tschacher, 2016). Although uni- and bi-directional types of interactions would help structure the collective rhythm, an emergent collective rhythm, as defined previously, can only arise from the mutual modulation observed during bi-directional coupling.

### The Social Effects of Interpersonal Coordination

Considering that the coordination of bodily states may facilitate or attenuate a shared experience and common timing with our conspecifics and may foster physiological homeostasis and emotional regulation within the individual, it is not surprising that it has been linked to different social effects (Figure 3), such as wellbeing, social cohesion and the feeling of connection (Wheatley et al., 2012; Prochazkova and Kret, 2017). Even though other mechanisms (e.g., shared purpose or intentions) may explain those social effects, our approach emphasizes that the spontaneous coordination of bodily states may increase the probability of experiencing them.

These positive effects have also been reported beyond dyads (von Zimmermann and Richardson, 2016; Jackson et al., 2018), particularly, physiological synchronization has been associated with group cohesion during, for example, teamwork or musical performance (Palumbo et al., 2016), and growing evidence in other social contexts suggests that this relationship is complex and non-linear (Palumbo et al., 2016; Wallot et al., 2016; Wood et al., 2018; Wiltshire et al., 2019; Mayo and Gordon, 2020; Dumas and Fairhurst, 2021; Hoehl et al., 2021). To the best of our knowledge, the only evidence of the prosocial consequences of mimicry at a group level is in studies of emotional contagion (e.g., Barsade, 2002), but they do not specifically tackle spontaneous mimicry. In non-human primates, some studies have related spontaneous facial mimicry to the development and maintenance of social bonds through social play (Palagi et al., 2019; Anderson and Kinnally, 2021). Likewise, whether the social consequences of behavioral mimicry also exhibit complex patterns at the group level has been little explored (see Hess, 2019).

Remarkably, the social effects that result from interpersonal coordination can also be accessed under artificial conditions (Figure 3A: artificial manipulation). In other words, once the inner workings of the coordination phenomenon are understood, humans can exert control over their own and others’ experiences of connection (Wheatley et al., 2012). Specifically, creating an artificial experience by setting off a coordinated activity has been shown to promote prosocial effects. For example, participants asked to move their bodies in synchrony show physiological linkage and report increased rapport (Lakin et al., 2003; Wheatley et al., 2012), even within an immersive virtual reality environment (Tarr et al., 2018). Likewise, manipulating the emotional content of an interaction can create affiliation and rapport between participants. Individuals primed to the concept of affiliation before a word scramble task have been shown to increase mimicry responses, particularly in contexts where the goal is to create rapport with others (Lakin and Chartrand, 2003). However, unlike synchrony, mimicry was found not to increase rapport or trust in virtual scenarios (Hale and Hamilton, 2016).

This artificial manipulation has also been shown to promote antisocial effects; for example, aggression, destructive obedience and reduced creativity and dissent have been observed after asking participants to perform synchronized activities such as walking or singing (Gelfand et al., 2020, but see Mogan et al., 2019). This artificial manipulation can even lead to a complete disruption of social effects, like when synchronization is inhibited by asking participants to follow a beat asynchronously (Hove and Risen, 2009). Nonetheless, while disrupting facial mimicry (e.g., holding a pencil in the mouth: Palagi et al., 2020; administration of hormones: Kraaijenvanger et al., 2017) hinders recognition of other’s facial and body expressions, it is not clear to what extent this affects affective bonds, empathy or the sense of belonging among interactants during naturalistic group activities.

In sum, the clearest picture we have of the social effects of interpersonal coordination through mimicry and synchrony is biased toward prosocial phenomena and dyadic interactions. Distinguishing the emotional and sociocultural constraints and contingencies at the individual level (e.g., levels of empathy, internal tempo) from those at the level of the interaction (e.g., emotional content, goal, complexity of the task) and at the social level (e.g., social niche) can help to deepen our understanding of this complex relationship and its characteristics in both natural and artificial settings, for example, by shedding light on whether the cultural encoding in the participants is accountable for the antisocial effects observed during artificial manipulations, or whether individual differences in internal tempo can impact the intensity of the social effects reported by the participants of an interaction.

## Discussion

### Collective Rhythm

Remarkable examples of the emergence of a collective rhythm are the activity cycles of ants, the shimmering waves propagated across the colony surface of giant bees, or the “Mexican wave” behavior performed by crowds in stadiums (Couzin, 2018). As a group level phenomenon, this rhythmicity could enhance the chances of survival and reproduction, such as in the giant honeybees that have been shown to use shimmering as a colony defense against hornets (Kastberger et al., 2008). As mentioned before, the capacity to influence and be influenced by others’ bodily states in non-intuitive, complex ways suggests that there is a possibility that during group dynamics a collective rhythm emerges from the temporal organization of behavior at the individual level.

Taking the evidence presented so far, in the sections below we explore some of the properties that could be associated with an emergent collective rhythm, and how this group-level phenomenon could be related to a collective feeling of wellbeing and connection. Additionally, we argue that this phenomenon could be artificially manipulated according to the sociocultural context, in a similar way as it has been reported for small-scale interactions.

#### The Properties of an Emergent Collective Rhythm

In general, very little has been explored about an emergent collective rhythm, but two approaches have given insights on its properties. On the one hand, the structural organization of some behaviors shows similar clustering across a wide range of time scales (e.g., conversation: Abney et al., 2014). This hierarchical clustering is observed commonly in language, where syllables are nested in words and words in sentences, sentences in interventions, and so on. Changes in speaking rate, for example, can affect how these events are clustered over time (Ramírez-Aristizabal et al., 2018). This has also been observed in, for example, the temporal pattern from seconds to hours of the locomotor activity of quails (Guzmán et al., 2017). Additionally, the pattern of resting periods of mice and healthy humans has been observed to be more nested than that of mice without a circadian clock gene or humans suffering from major depressive disorders (Nakamura et al., 2008). Likewise, the pattern of acoustic events of interactions between either speakers, musicians or killer whales is more nested than their individual rhythmic patterns (Kello et al., 2017). If we consider that the nested organization of spontaneous behaviors is found in several species, and that it is greater in healthy individuals and in contexts in which several individuals are involved, it is reasonable to expect that a collective rhythm emerging from group activities will exhibit a nested clustering of behavioral onsets at different time scales. However, more research is needed on this global phenomenon in group activities.

On the other hand, some behaviors are characterized by a lower variability than that observed in each participant’s movements, i.e., dimensional compression (Riley et al., 2011; Fusaroli et al., 2014; Nowak et al., 2017). This reduced dimensionality of individuals’ movements or synergies has been observed in the temporal pattern of speech/pause dynamics during conversation (Fusaroli and Tylén, 2016) or in the walking direction and the common speed that emerge in pedestrians (Kiefer et al., 2017). In the case of a collective rhythm, this reduced variability could be interpreted as a more predictable rhythmicity, and its presence could signal affiliation (e.g., Fawcett and Tunçgenç, 2017) or could facilitate the interpersonal coordination of observers, as a steady pulse does in music. In any case, more research is needed on these topics.

Research on collective rhythm would benefit if instead of equating social coordination to the concept of synchronization and considering dynamics as an endless alignment between systems, more complex and multilayered dynamics were included (Fusaroli et al., 2014; Wood et al., 2018; Clayton et al., 2020). Social coordination may include, for instance, compensatory exchanges (e.g., dancing partners movements) where the behavioral repertoire of an individual limits the set of behaviors that another may adopt (e.g., Wallot et al., 2016) or intermittent coordination, where people move in and out of coordinated states (Dahan et al., 2016; Nowak et al., 2017; Mayo and Gordon, 2020), such as the mother-infant interactions, which tend to include short periods of shared emotional activity as well as states where one or both partners show no interest in interacting with the other (see Kokkinaki et al., 2017); or scenarios in which social exchanges over time are characterized by qualitatively distinct phases of coordination (i.e., phase transitions), such as collaborative problem solving (Wiltshire et al., 2018) or psychotherapy (García and Di Paolo, 2018). More research in this area could help us understand to what extent these dynamics have confounded synchronization experiments in the literature and, more importantly, what is their contribution to the rhythmic pattern at the group-level: do the mismatched emotional states in mother-infant dyads have fractal-like temporal structure? Are the different phases of coordination characterized by different patterns of dimensional compression?

#### Relationship to a Collective Feeling of Wellbeing and Connection

To our knowledge, there is no experimental evidence confirming how an emergent collective rhythm could be related to a collective feeling of wellbeing and connection, only correlational evidence found in the anthropological literature between collective activities and group cohesion, ecstasy, wellbeing, and solidarity (reviewed in Gelfand et al., 2020), such as marching together during military drills (McNeill, 1997: 2), the church services in early Christian practices (Ehrenreich, 2007: 65) or secular festivities during the Middle Ages (Ehrenreich, 2007: 92).

Confirmation of an emergent collective rhythm and its relationship to a collective sense of wellbeing could lead to a more straightforward explanation to the previously suggested association (Haidt et al., 2008; Wheatley et al., 2012; Mogan et al., 2017; Wood et al., 2018; Clayton et al., 2020) between collective coordination and what Emile Durkheim coined as “collective effervescence,” i.e., the feeling of belonging and assimilation experienced during collective rituals (cited in Xygalatas et al., 2011). Additionally, evidence in this regard would be compatible with the “hive hypothesis” (Haidt et al., 2008: 136) which builds on Durkheim’s collective effervescence and states that “people need to lose their selves occasionally by becoming part of an emergent social organism (…) and in which self-consciousness is greatly reduced and one feels merged with or part of something greater than the self.” Put differently, group dynamics could provide a common beat to which to coordinate, thereby overcoming individual rhythms (Wheatley et al., 2012). According to the evidence presented so far, a collective rhythm analogous to a musical beat would emerge from, at least, the accommodation of bodily states, would disperse depending on the participants’ interconnection structure (either by sensory perception, affiliative connection, cultural preferences) and, analogous to dyadic interactions, would facilitate feelings of connection and assimilation to the group, and contagious euphoria.

Evidence supporting these hypotheses comes from a pair of studies of a fire-walking ceremony in a Spanish village (Konvalinka et al., 2011; Xygalatas et al., 2011). The results show that, compared to non-related pairs, the heart rates of firewalkers and related spectators share similar temporal dynamics, such as a more structured pattern and a peak distributed around the fire-walk. Moreover, this shared pattern extended through a network from related to unrelated performers. These findings suggest that during a collective ritual, the coordination of bodily states may be constrained by the social network of the participants and that the resulting rhythmicity will relate to the activities that take place in it. Although only a few studies have addressed the influence of the network topology on the collective rhythm, they have shown that the level of coordination varies depending on the pattern of interconnections (i.e., topology) among participants (van de Rijt, 2018), and their internal tempo (Alderisio et al., 2017). Methods specifically designed to capture changes in network modularity over time could help elucidate, for example, whether the complementary dynamics observed during joint action (e.g., Wallot et al., 2016) can be seen as a small-scale instance of a modular organization subjected to certain environmental constraints and contingencies (see Bourbousson and Fortes-Bourbousson, 2016; Mayo and Gordon, 2020), and to what extent the intermittent coordination that results from such modularity contributes to the complexity and fluency of the collective temporal pattern.

Confirmation on the relationship between an emergent collective rhythm and a collective feeling of wellbeing could be of clinical relevance, as some disorders or impairments might target different aspects of the causal model presented in Figure 3. For instance, patients with autism spectrum disorder (i.e., endogenous rhythms: Bobin-Bègue, 2019) and people with decreased complexity of locomotion due to aging (i.e., locomotor rhythms: Almurad et al., 2018) have disruptions of individual rhythms. People with schizophrenia (Varlet et al., 2012) and social anxiety disorder (Varlet et al., 2014) have shown disrupted coordination dynamics in leader-follower interactions when the patient had to lead the coordination, but unaffected dynamics in unintentional coordination. Understanding how the collective rhythm emerges from the constraints and contingencies at different levels (e.g., individual vs. interaction level) could help develop intervention strategies that include not only patients but the people interacting with them, to improve their social exchange. For example, in addition to protocols that help patients with social anxiety disorder to manage their leader position during an interaction, these patients could benefit from social exchanges where no agent controls the social dynamic, i.e., the type of interaction that could lead to an emergent collective rhythm. Or patients with disruptions of locomotor rhythms could benefit from group activities where they synchronize their movements to evenly spaced rhythms.

#### Artificial Manipulation of the Collective Rhythm

It remains to be seen whether a collective rhythm emerges during group activities and to what extent it is related to the collective effervescence experience and the hive hypothesis. However, a plausible example of the artificial manipulation of the collective rhythm according to the sociocultural context, is the link between ritualistic synchrony and the theory of cultural evolution known as tightness-looseness theory (Gelfand et al., 2020). According to this theory, in order to survive, societies marked by higher rates of socioecological threats like natural disasters or food insecurity tend to develop tighter cultural norms than those with fewer coordination needs. In that particular context, the effects of ritualistic synchrony would be adaptive, given that in the face of greater threats, the benefits (i.e., cooperation, coordination, cohesion) of synchronized activities such as dancing, chanting or marching, would outweigh the negative effects (e.g., less creativity) associated with them. Within this framework, Gelfand et al. (2020) predict that the use of synchrony in ritualistic scenarios would be common after periods that require social coordination, such as after ecological or social threat. This prediction needs to be further investigated, but it is concordant with the argument that humans artificially manipulate the effects of interpersonal coordination at a collective level according to the needs of the sociocultural context.

Overall, research on this topic would help to explain anthropological research (reviewed in McNeill, 1997; Ehrenreich, 2007; Haidt et al., 2008) showing, for instance, that although the degree of synchronized physical activity observed changes over the years and between social groups, the feeling of connection remains; it could also improve our knowledge of derived human traits with fixed (e.g., music) and labile (e.g., storytelling) tempo.

### Concluding Remarks

During group activities, the interpersonal coordination at different levels and modalities gives a temporal structure to the collective dynamics. The evidence suggests that this phenomenon facilitates the emergence of a collective rhythm, i.e., temporal regularities that cannot be predicted from the individual periodicities, and that this group-level property could be related to a collective feeling of wellbeing and connection. Although more research is needed to test this argument, we highlight two ways of obtaining a clearer picture of the complexity associated with social coordination at the collective level. First, it is important to take into account that behavioral dynamics go beyond simple synchronization. Developing methods, including experimental settings, that explore complex and multilayered social dynamics with, for example, compensatory exchanges or intermittent coordination, may clarify their influence on the construction of a collective rhythm and its consequences.

Second, our framework naturally incorporates the variability of the social consequences of interpersonal coordination. We suggest that the study of the social effects of interpersonal coordination should highlight that in the early stages of life these are important for the survival, homeostasis, and adaptation of the child to a dynamic physical and social environment. Later in life, it should be emphasized that these effects are shaped by the constructed niche (biological and sociocultural) in which the individual developed and the social constraints in which the interaction takes place. Overall, our understanding of how these evolutionary social effects of interpersonal coordination have been adapted to the constructed environment would be improved with studies in which artificial experiments (e.g., finger tapping) are applied to non-WEIRD participants (i.e., people from Western, educated, industrialized, rich and democratic societies), and with studies in more naturalistic settings that include the variety of cultural practices in which social coordination fosters feelings of cohesion and collective effervescence.

## Author Contributions

AF and GR-F jointly conceived, designed, and revised the manuscript. AF wrote the first draft of the manuscript. Both authors contributed to the article and approved the submitted version.

## Conflict of Interest

The authors declare that the research was conducted in the absence of any commercial or financial relationships that could be construed as a potential conflict of interest.

## Publisher’s Note

All claims expressed in this article are solely those of the authors and do not necessarily represent those of their affiliated organizations, or those of the publisher, the editors and the reviewers. Any product that may be evaluated in this article, or claim that may be made by its manufacturer, is not guaranteed or endorsed by the publisher.
